# Megalencephaly Syndromes: Exome Pipeline Strategies for Detecting Low-Level Mosaic Mutations

**DOI:** 10.1371/journal.pone.0086940

**Published:** 2014-01-31

**Authors:** William J. Tapper, Nicola Foulds, Nicholas C. P. Cross, Paula Aranaz, Joannah Score, Claire Hidalgo-Curtis, David O. Robinson, Jane Gibson, Sarah Ennis, I. Karen Temple, Andrew Collins

**Affiliations:** 1 Department of Human Genetics and Genomic medicine, Faculty of Medicine, University of Southampton, Southampton, Hampshire, United Kingdom; 2 Wessex Regional Genetics Laboratory, Salisbury District Hospital, Salisbury, Wiltshire, United Kingdom; 3 UHS NHS Foundation Trust and Department of Human Genetics and Genomic Medicine, Faculty of Medicine, University of Southampton, Southampton, Hampshire, United Kingdom; 4 Department of Genetics, School of Sciences, University of Navarra, Pamplona, Spain; University of Bonn, Institut of Experimental Hematology and Transfusion Medicine, Germany

## Abstract

Two megalencephaly (MEG) syndromes, megalencephaly-capillary malformation (MCAP) and megalencephaly-polymicrogyriapolydactyly-hydrocephalus (MPPH), have recently been defined on the basis of physical and neuroimaging features. Subsequently, exome sequencing of ten MEG cases identified *de-novo* postzygotic mutations in *PIK3CA* which cause MCAP and *de-novo* mutations in *AKT* and *PIK3R2* which cause MPPH. Here we present findings from exome sequencing three unrelated megalencephaly patients which identified a causal *PIK3CA* mutation in two cases and a causal *PIK3R2* mutation in the third case. However, our patient with the *PIK3R2* mutation which is considered to cause MPPH has a marked bifrontal band heterotopia which is a feature of MCAP. Furthermore, one of our patients with a *PIK3CA* mutation lacks syndactyly/polydactyly which is a characteristic of MCAP. These findings suggest that the overlap between MCAP and MPPH may be greater than the available studies suggest. In addition, the *PIK3CA* mutation in one of our patients could not be detected using standard exome analysis because the mutation was observed at a low frequency consistent with somatic mosaicism. We have therefore investigated several alternative methods of exome analysis and demonstrate that alteration of the initial allele frequency spectrum (AFS), used as a prior for variant calling in samtools, had the greatest power to detect variants with low mutant allele frequencies in our 3 MEG exomes and in simulated data. We therefore recommend non-default settings of the AFS in combination with stringent quality control when searching for causal mutation(s) that could have low levels of mutant reads due to post-zygotic mutation.

## Introduction

Megalencephaly syndromes (MEG) have been noted to arise as sporadic overgrowth disorders and have been associated with variable additional malformations including cortical dysplasia, developmental vascular anomalies, distal limb malformations and variable connective tissue dysplasias [1]. Recent findings led to the characterisation of two MEG syndromes on the basis of physical and neuroimaging anomalies [1]. These disorders are:

1. Megalencephaly-capillary malformation syndrome (MCAP), for which most patients would previously have been classified as Macrocephaly-capillary malformation syndrome (MCM).
2. Megalencephaly-polymicrogyriapolydactyly-hydrocephalus syndrome (MPPH), which is closely related to MCAP but patients lack vascular malformations and syndactyly.

Subsequently, exome sequencing of 10 affected individuals, 8 showing the MCAP phenotype, one with MPPH and one with an overlapping phenotype, was used to identify *de-novo* germline and postzygotic mutations in *AKT3*, *PIK3R2* and *PIK3CA* which are responsible for the MCAP and MPPH phenotypes [2]. In this paper, the authors noted that patchy skin vascular malformations and asymmetric overgrowth seen in MCAP were consistent with the presence of postzygotic mosaicism being present in some of the cases. In the case of syndromes for which mosaic skin defects are a feature, the concept of a lethal gene surviving by mosaicism was first suggested in 1987 [3]. Such mutations would lead to early embryonic death when present in the zygote but mutated cells could survive if in close proximity to normal cells and the mutation could arise, for example, as an early somatic mutation. Subsequently the Proteus overgrowth syndrome was found to be caused by a somatic activating mutation in the *AKT1* gene proving the hypothesis of somatic mosaicism [4]. The authors identified tissues and cell lines from patients with Proteus syndrome as having admixtures of mutant alleles ranging from 1%–50%.

The analysis of a family trio showing clinical features overlapping MCAP and MPPH by Riviere et al [2] initially identified a *de-novo* mutation in the *AKT3* gene and they identified one other individual with such a mutation establishing this as a rare cause of megalencephaly. Recognising *AKT3* as a major downstream regulator of PI3K signalling they directed their focus on genes within this pathway to explain the megalencephaly phenotype. They subsequently identified one recurrent mutation in *PIK3R2* (c.1117G>A, p.G373R) in three siblings affected with MPPH arising as a *de-novo* germline mosaic in one parent. The same variant was identified in a further 10 cases with MPPH.

By reducing the stringency of their standard exome analysis pipeline so that variants with just two or more mutant reads were considered, the authors identified several different *de-novo* mutations in *PIK3CA* as causes of MCAP. These variants, which include c.2176G>A p.E726K, have mutant allele frequencies ranging from 2–50% in their exome data and were missed by their standard exome analysis pipeline which used the default hard filtering parameters [5].

We exome-sequenced three sporadic MEG cases and searched for genes with recurrent *de-novo* mutations. Due to the genetic heterogeneity of these MEG cases, and the involvement of post-zygotic mutations with low mutant allele frequencies, our standard analysis failed to identify any genes with recurrent *de-novo* mutations. We therefore optimised our exome analysis pipeline for the detection of variants with low mutant allele frequencies and reanalysed our MEG exomes. This revised analysis identified the underlying disease mutations in all three of our MEG cases. We describe here the phenotypes and causal mutations of our patients, their correspondence with the newly defined MCAP and MPPH syndromes, and an evaluation of the methods used to optimise our pipeline for the identification of variants with low mutant allele frequencies. These findings should inform optimisation analysis pipelines for detecting low level mosaic causal variants in next generation sequencing data for these and other diseases.

## Materials and Methods

### Cases and Phenotypes

Three MEG cases were selected for exome sequencing whose phenotypes according to proposed diagnostic criteria [6] are listed in Table 1. All three cases show characteristic MRI scans with varying degrees of cortical dysplasia. Two patients have arrhythmias very characteristic of MEG. Patient 1 has macrocephaly (+8SD), a broad forehead, a wide flat nasal bridge, downslanting palpebral fissures, a philtral heamangioma, lax abdominal wall, truncal hypotonia and small joint hypermobility. She also has widespread keratosis pilaris especially on her limbs and deep palmar and plantar creasing. Ulnar deviation at the wrists was noted, very much like that seen in Costello syndrome. Hydrocephalus requiring a third ventriculostomy and shunt insertion developed with time and on the last genetics assessment, aged 5 years, moderate/severe developmental delay in all areas was apparent. She has had a normal array-CGH and is normal for PTEN (data not shown). Patient 2 had macrocephaly (+4SD), characteristic facies with a broad forehead hypertelorism and a marked philtral haemangioma. He also had widespread cutis marmorata and 2.3 toe syndactyly. A pernicious atrial arrhythmia developed at 2 months of age for which treatment was unsuccessful and the patient died. No developmental information is therefore available on this case. A karyotype showed a normal male complement. Patient 3 has macrocephaly (+6SD which had been noted initially antenatally on the 20 week scan), characteristic facies with a philtral haemangioma and widespread cutis marmorata in the newborn period. He was last reviewed by genetics at 2 years of age at which point he had moderate motor delay, but borderline normal cognitive development. He was noted to have keratosis pilaris of all four limbs and deep palmer and planter creases. His general health had been good other than one renal tract infection at 5 months of age that had resulted in some renal scarring. Array-CGH was normal.

**Table 1:** Causal genotype and phenotypes in three cases with megalencephaly.

Causal mutation	Patient 1	Patient 2	Patient 3
	*PIK3CA* p.E726K	*PIK3CA* p.E726K	*PIK3R2* p.G373R
Major phenotypic criteria (requires one)			
Macrocephaly	+++	++	++
Capillary malformation	+++	+++	++
Overgrowth/asymmetry	+++		+
Ventriculomegaly	+++		+
Cavum septum pellucidum or cavum vergae			
Cerebellar tonsillar herniation	+	Not tested	
Cerebral and/or cerebellar asymmetry	++	Not tested	+
Minor phenotypic criteria (requires two)			
Developmental delay	++	Not tested	+
Midline facial capillary malformation	++	+	++
Neonatal hypotonia	+++	+	+
Syndactyly or polydactyly		+	
Frontal bossing	+	+	+
Connective tissue abnormality	+		+
Hydrocephalus	+		

Severity of phenotype indicated by number of +’s.

Blank cells represent absence of phenotype.

### Exome Sequencing and Standard Analysis Pipeline

Genomic DNA from the three MEG patients was extracted from whole blood and their exomes were enriched and captured using Agilent SureSelect kit (Human All Exon 50 Mb) and sequenced using an Illumina HiSeq 2000 at the Wellcome Trust Centre for Human Genetics at Oxford. Approximately 6 gigabases of 100bp paired-end read sequences were generated per sample. The Novoalign software (version 2.08.02, Novocraft Technologies, Selangor, Malaysia) was used to align this sequence data to build 36 (hg18) of the reference human genome. Duplicate reads, due to PCR clonality or optical duplicates, reads mapping to more than one location, and reads failing platform or vendor quality checks were removed. Coverage statistics were calculated using the BedTools software.

In the standard analysis pipeline, variants were called on a single sample basis using a two-step procedure in Samtools version 0.1.18 [7]. In the first step mpileup is used to compute the likelihood of data given each possible genotype. During this process probabilistic realignment is used to compute the base alignment quality (BAQ), which represents the probability that a read base is mis-aligned. In general, this increases sensitivity and decreases specificity and helps to exclude false positives due to alignment artifacts caused by nearby indels. In the last step, the view command of bcftools is used to call variants when the variant calling threshold (P) is below 0.5, P(ref|D,F) and where ref is the reference allele, D are data and F is the prior allele frequency spectrum (AFS). The AFS describes the distribution of SNP sites based on the number of chromosomes that carry a given allele in a sample [8] and is computed using an iterative EM algorithm. In the standard analysis pipeline the ‘full’ AFS model is used to compute the probability of seeing *k* alternative (non-reference) alleles from *M* chromosomes tested.

### Alternative Exome Pipelines

We explored several non-standard pipelines to determine which provides greater power to detect variants with low mutant allele frequencies which may result from post zygotic mutations. These alternative methods are outlined as follows:

1. Multi-sample calling of all three MEG cases simultaneously using Samtools mpileup. By combining evidence across samples this method can identify variants which have insufficient data in individual samples due to low coverage. It is also possible that some false positive variants will be excluded when multiple samples are used. However, the power to detect singleton SNPs is diminished when multiple samples are considered.
2. Varying the initial AFS using bcftools. If the initial AFS is far from the solution EM iteration may require more cycles and, more importantly, may converge to a local, sub-optima, solution [7]. The final SNP calling outcomes may therefore be sensitive to the choice of the initial AFS. Li (2010 http://lh3lh3.users.sourceforge.net/download/multigeno.pdf) describes two alternative initial AFS conditions, ‘cond2’ and ‘flat’, derived from the infinite site Wright-Fisher model. These alternatives compute the probability of seeing *k* non-reference alleles from two chromosomes (cond2) or from one chromosome (flat) which gives a flat initial AFS. We therefore recalled variants using the cond2 and flat initial AFS settings.
3. Reduced variant calling threshold using bcftools. In the standard pipeline, variants are called when the variant threshold is less than 0.5. Using bcftools (view option –p), we relaxed this threshold to 0.75 and 1.
4. Elevation of the mutation rate using bcftools. In the standard pipeline, the scaled mutation rate is 0.001. We used bcftools (view option –t) to increase this to 0.01 and 0.1.

### Simulation of Sequence Data with a Range of Mutant Allele Frequencies

To further explore the sensitivity of these methods to detect variants with low mutant allele frequencies we simulated 100bp paired end sequence data with total read depth ranging from 4 to 100 and mutant read frequencies ranging from 1% to 50%. All reads were simulated to be mapped in the correct orientation and within insert size, to be evenly distributed on the positive and negative strand, to have mapping qualities of Phred 60, and to have base qualities of Phred 30. Reads with the reference allele were identical to the reference (CIGAR = 100 M, MD:Z:100) while reads with the alternative allele had one mismatch that was located in the middle of the read (CIGAR = 100 M, MD:Z:50G49). The sensitivity of each method of exome analysis was determined by recording the minimum alternate allele frequency at which the variant was detected and Wilcoxon signed rank tests were used to compare the minimum alternate allele frequency between methods.

### Variant Filtering and Comparison between Patients

Following variant calling, vcfutils which is part of VCFTools [9] and custom scripts were used to select variants with good supporting evidence and to stratify them into two tiers according to a range of filtering criteria. In the first tier, vcfutils was used to select variants with base call accuracy ≥90% (Phred ≥10), total read depth ≥4, alternative read depth ≥2, strand bias P≥0.0001, base quality bias P≥1e-100, tail bias P≥0.0001, and HWE P≥0.0001. In addition, custom scripts were used to exclude variants located within or adjacent to repetitive DNA including homopolymers ≥5 bp. In the second more stringent tier variants were required to have ≥3 reads with the alternative allele, ≥1 read with the alternative allele on each strand, strand bias P≥0.01, base quality bias P≥0.01, tail bias P≥0.01, mapping quality bias P≥0.01, and excluding genes with excessive numbers of pathogenic mutations due to their presence within highly polymorphic regions of the genome, assembly misalignment, or errors in the reference genome [10].

Novel variants were identified by using Annovar [11] filtering against public databases (dbSNP129, dbSNP132, 1000 genomes phase 1 July 2010 release, and European American exomes from the NHLBI Exome Sequencing Project) and an in-house database (n = 100 exomes) of known variants. To generate a list of candidate genes, the novel variants in each patient were compared to identify genes which contain novel variants in two or more patients which is a common strategy of exome analysis.

### Mutation Validation by Sanger Sequencing and Pyrosequencing

Sanger sequencing of PCR amplicons from whole blood-genomic DNA in the probands and two of three unaffected pairs of parents was used to confirm the presence of variants identified *in silico* by exome sequencing and to determine their *de-novo* status. For the *PIK3CA* variant (c.2176G>A, p.E726K) pyrosequencing was used to determine the level of mosaicism in whole blood-genomic DNA samples from the probands and their parents by calculating the average mutant allele frequency from two estimates. Although the level of PIK3CA mosaicism is higher in skin fibroblasts and saliva blood was used since saliva and skin fibroblasts were unavailable.

Whole blood-genomic DNA samples from the two probands and their parents were amplified using primers 5′-biotin-TTGCACGATTCTTTTAGATCTGAG-3′ (forward) and 5′-TCCAAATCCTAATCTGCTTGATTC-3′ (reverse) and sequenced with the primer 5′-TCACACACCTTTTGTGTT-3′ (reverse orientation). Pyrosequencing reactions were performed according to the manufacturer's instructions using the PSQ 96 single nucleotide polymorphism (SNP) Reagent Kit (Biotage), which contained the enzyme and substrate mixture and nucleotides. Two independent estimates were made for each case.

## Results

The coverage statistics show that the mean depth per sample ranged from 46 to 52 reads and that 85 to 87% of the Gencode defined target bases were covered by 10 or more reads. Full coverage information is available in Supplementary .

### Identification of Causal Variants in PIK3CA and PIK3R2

We examined exome data from three MEG cases for the mutations in *AKT3*, *PIK3R2,* and *PIK3CA* which had been identified as causal [2]. All methods used correctly identified the causal variant in patient 1, who is heterozygous for the *PIK3CA* c.2176G>A, p.E726K variant, and in patient 3 who is heterozygous for the *PIK3R2* c.1117G>A, p.G373R variant (Table 2). In patients 1 and 3, the alternative allele accounted for a large proportion of the total reads, between 42% (15/36) and 48% (13/27), which enabled a high base call accuracy (Phred 155–185) and confident inference of the heterozygous genotype (Phred 99).

**Table 2:** Characteristics of causal variants in *PIK3CA* and *PIK3R2.*

Feature	Patient 1	Patient 2	Patient 3
Causal mutation	*PIK3CA* p.E726K	*PIK3CA* p.E726K	*PIK3R2* p.G373R
Frequency of mutant allele by pyrosequencing	20%	18%	NA
Total number of reads	36	36	27
Frequency of reference allele reads	58.3% (8+,13−)	88.9% (13+,19−)	51.9% (6+,8−)
Frequency of mutant allele reads	41.7% (4+,11−)	11.1% (1+,3−)	48.1% (9+,4−)
Mapping quality	60	60	60
Phred scaled genotype likelihoods (00/01/11)	185,0,236	12,0,233	179,0,197
Default (Phred base accuracy, genotype confidence)	155, 99	Not identified	149, 99
Allele frequency spectrum = Flat	185, 99	12.3, 15	179, 99
Allele frequency spectrum = Cond2	183, 99	10.6, 14	177, 99
Mutation rate = 0.01	165, 99	Not identified	159, 99
Mutation rate = 0.1	176, 99	4.57, 12	170, 99
Variant threshold = 0.75	155, 99	Not identified	149, 99
Variant threshold = 1.0	155, 99	0.0684, 7	149, 99
Multi-sample default	157, 99	157, 10	139, 99

In patient 2, the causal allele (*PIK3CA* c.2176G>A, p.E726K) was present at a much lower read frequency, accounting for just 11% (4/36) of the total reads. As a result the default pipeline determined that patient 2 was homozygous for the reference G allele despite the genotypic Phred scaled likelihoods identifying the heterozygous genotype as the most likely and the reference homozygous genotype having a low probability (P = 0.063, Phred = 12). The correct heterozygous genotype for this variant was identified only when the flat and cond2 AFS settings were used, when the scaled mutation rate was ≥0.1, when the variant threshold is set to 1, or when the samples were analysed together. The strongest evidence for the variant was obtained when the initial AFS was set to flat which gave a base call accuracy of 94.1% (Phred = 12.3) and a genotype quality of 96.8% (Phred = 15) (Table 2). The cond2 model gave similar but slightly less significant results. Although the variant was also called when the mutation rate and variant threshold were substantially increased, the resulting base call accuracies of 65.1% (Phred = 4.57) and 1.6% (Phred = 0.0684) respectively, are below our variant filtering threshold of 90% (Phred≥10) so they would normally be excluded from the results.

To explore the sensitivity of the multi-sample method for identifying variants with low alternate allele frequencies, we repeated this analysis using two copies of patient 2, which have mutant allele frequencies of 11% for the *PIK3CA* variant (c.2176G>A, p.E726K), and patient 3 which has no evidence for this variant. This analysis failed to identify the variant which demonstrates that, in these data, multi-sample calling can only detect low frequency variants when at least two of the samples being tested have the same variant and it has a high alternate allele frequency in at least one of these samples.

### Confirmation of the Causal Variants and their Correspondence with MCAP and MPPH

The presence of the causal variants in all three of our MEG cases was confirmed by Sanger sequencing. Sanger sequencing was also used to determine that the *PIK3CA* variant (c.2176G>A, p.E726K) in patients 1 and 2 is *de novo*. We could not determine the *de novo* status of the *PIK3R2* variant (c.1117G>A, p.G373R) in patient 3 since parental DNA was unavailable. In patients 1 and 2, the frequency of the mutant allele was estimated to be 20 and 18% respectively using pyrosequencing (Table 2).

The *PIK3R2* variant in patient 3 has previously been associated with the MPPH phenotype which resembles MCAP but lacks vascular malformations and syndactyly [2]. The phenotypes defined for this patient (Table 1) shows an absence of syndactyly, but there is evidence for capillary malformations which contradict one of the phenotypic characteristics of MPPH. In addition this patient also has significant bifrontal band heterotopia which is inconsistent with MPPH. A close relationship between these forms of MEG has been noted including a case with strongly overlapping phenotypes [2]. As more cases of MEG are reported it is likely that further phenotypic overlaps will become apparent.

Of 24 cases with *PIK3CA* mutations, 23 were diagnosed with MCAP [2]. Accordingly, patients 1 and 2 would be classified as MCAP rather than MPPH due to their *de-novo* mutations in *PIK3CA*. The phenotypes of patients 1 and 2 are consistent with the published definition of MCAP [1] although the phenotypic data for patient 2 is limited due to the patient’s short lifespan.

### Exome Wide Evaluation of Our Standard and Alternative Exome Analysis Pipelines

To determine the effects of the alternative variant calling methods on the number of variants identified and their data quality we applied these methods to the three MEG exomes and filtered the variants using the moderate (tier 1) and stringent (tier 2) quality criteria. We then identified variants that were ‘unique’ to each of the alternative methods in comparison with the default analysis. These unique variants were characterized by calculating various indicators of sequence and data quality such as the average base call accuracy, the proportion of variants with significant biases (strand, base quality, tail, or mapping quality bias P≤0.05), and the proportion of variants which occur in genes with excessive numbers of pathogenic mutations. Finally, novel variants were identified and compared between samples to identify genes which contain novel variants in two or more samples.

In terms of the average number of variants in tier 1, our default analysis was the most conservative (n = 22,275 variants) and the multi-sample method was the most liberal (n = 23,934) (Table 3). Although the multi-sample method identified the most variants it was also the only method which did not identify all of the variants from the default analyses. On average, 378 of the default variants were not called by the multi-sample approach. This group of variants has the highest proportion of variants located in genes with excessive mutation rates (33.6% versus 13.4%) and variants with significant biases (47.4% versus 11.5%) which suggests that many of them are likely to be false positives (Supplementary Table S2). When the variant threshold was altered no additional variants were identified in comparison to the default analysis. As a result the variant threshold options were excluded from further analyses.

**Table 3:** Mean number of variants identified.

Method of exome analysis	Mean number of variants		Mean number of novel variants	
	Tier 1	Tier 2	Tier 1	Tier 2
Default	22275	16223	191	103
Allele frequency spectrum = Flat	23558	16267	697	109
Allele frequency spectrum = Cond2	23460	16265	638	108
Mutation rate = 0.01	22686	16243	278	104
Mutation rate = 0.1	23134	16257	467	106
Variant threshold = 0.75	22275	16223	191	103
Variant threshold = 1.0	22275	16220	191	103
Multi-sample default	23934	15928	205	99

In comparison to the default analysis, the unique variants identified by the AFS options (flat and cond2), mutation rates (0.01 and 0.1), and multi-sample analysis have significantly higher proportions of variants with only two alternate alleles and variants with alternative alleles on only one strand (Supplementary table S2). In addition, these unique variants have lower base call accuracies and fewer reads (total and alternative) compared with the default analysis which suggests that many of them may be false positives. However, the causal variant in *PIK3CA* with low copy number is amongst these unique variants so hard filtering must be approached with caution when low copy number mosaic mutations are considered.

When tier 1 filtering is used to identify genes with novel variants in two or more patients, the causal gene for MCAP (*PIK3CA*), shared by patients 1 and 2, is not identified by the default analysis but is correctly called among 50 genes identified by the multi-sample method and 143 and 169 genes identified by the AFS cond2 and AFS flat models respectively (Table 4). Ten of these genes have been identified as having abnormally high mutation rates [10] so are unlikely to contain causal variation but a significant number of genes remain when these are excluded. When these comparisons were repeated using the more stringent tier 2 filtering, the default analysis identified five genes (*DIP2C*, *TP53BP1*, *PLEKHG5*, *BAHCC1*, and *KIAA1324*) with novel variants in two patients (Table 4) but failed to identify the *PIK3CA* mutations shared by patients 1 and 2. In contrast, the alternative AFS methods (flat and cond2) identified one additional gene with novel variants in two patients which was *PIK3CA*. The multi-sample method also identified these genes as being present in twopatients in addition to one other gene in two patients (*TTLL5*) and two other genes (*AURKC* and *NPEPL1*) in three patients.

**Table 4:** Number of genes with novel variants in x or more samples.

	Moderate QC, tier 1			Stringent QC, tier 2		
Number of samples	≥1	≥2	≥3	≥1	≥2	≥3
Default	536	12	1	297	5	0
AFS Flat	1768	169	12	312	6	0
AFS Cond2	1650	143	7	310	6	0
Mutation rate 0.01	771	26	1	301	5	0
Mutation rate 0.1	1244	87	2	307	5	0
Multi-sample default	527	50	15	279	9	2

The variants in *DIP2C* were excluded after Sanger sequencing confirmed their presence in parents who have normal phenotypes. The other genes identified by our default and alternative analyses were also ruled out on the basis that they involved at least one synonymous variant or one variant that was predicted to be benign (Supplementary Table S3).

**Figure 1:**
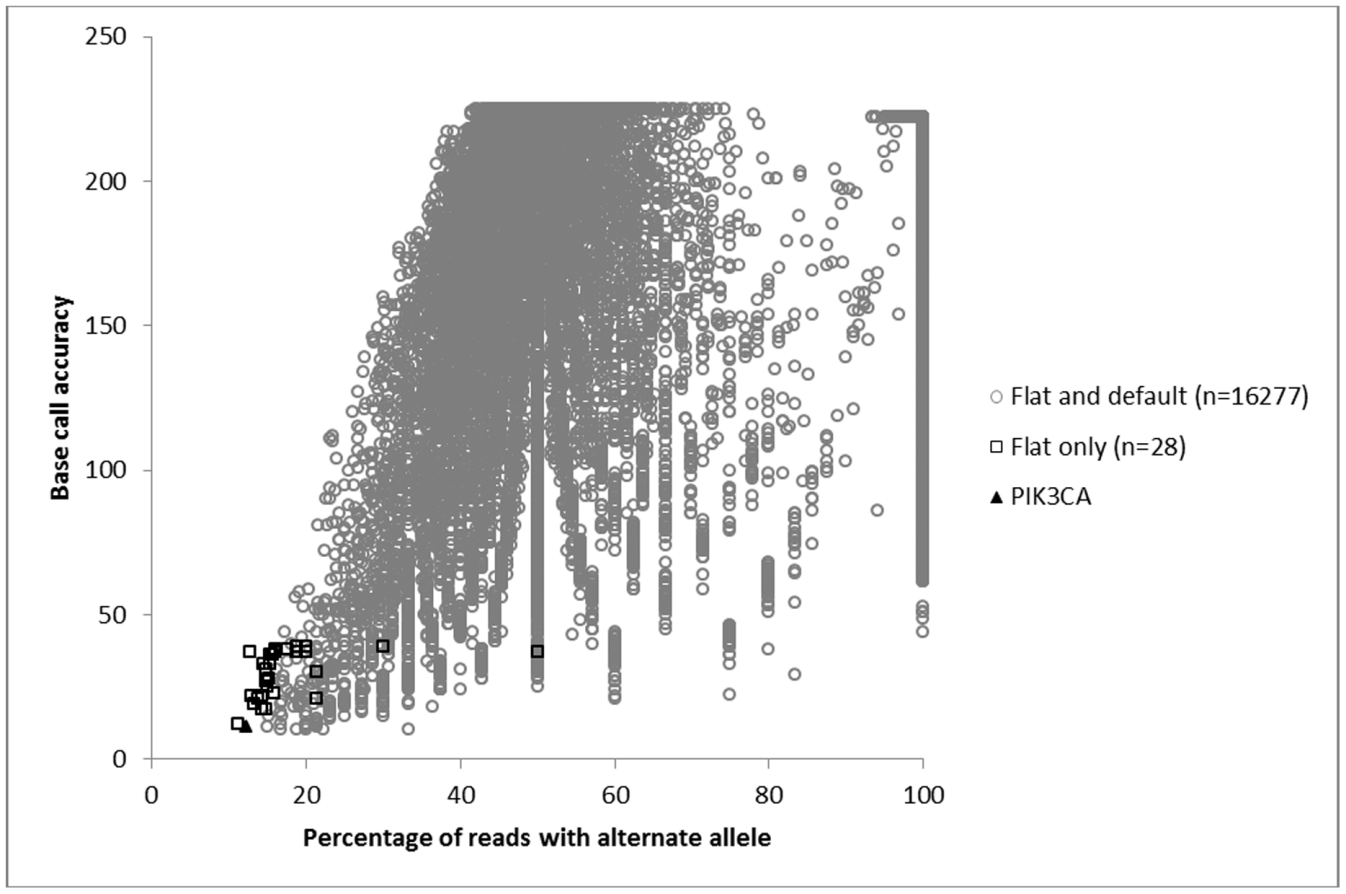
Variants identified in sample 2 by the full and flat AFS models including the mosaic post zygotic variant in *PIK3CA*.

When focusing on the variants in tier 2 which pass stringent QC, the methods used for variant calling are very similar in terms of the average numbers of variants identified which is 16203 per sample (Table 3). However, only three of these methods (AFS flat and cond2 and multi-sample) identified the causal *PIK3CA* variant when it was present at a low mutant allele frequency in patient 2. Of these methods, the flat AFS model gave the strongest support for the low frequency *PIK3CA* variant and fewer candidate genes with recurrent novel mutations compared with the multi-sample method. Furthermore, the multi-sample method could only detect the low frequency *PIK3CA* variant when the same variant was present in a second sample with high mutant allele frequency. In comparison to the default analysis, the flat AFS model identified an average of 44 additional variants per sample which includes the causal *PIK3CA* variant in patient 2 (Supplementary Table S4). These additional variants have relatively low base call accuracies (Phred≤39) and 84% of them have mutant allele frequencies ≤20%. As a result the majority of these variants form a cluster which partly overlaps with the lowest level mutations that are called by the default analysis (Figure 1 and supplementary Figures S1 and S2).

### Evaluation of Exome Analysis Pipelines Using Simulated Data

**Figure 2:**
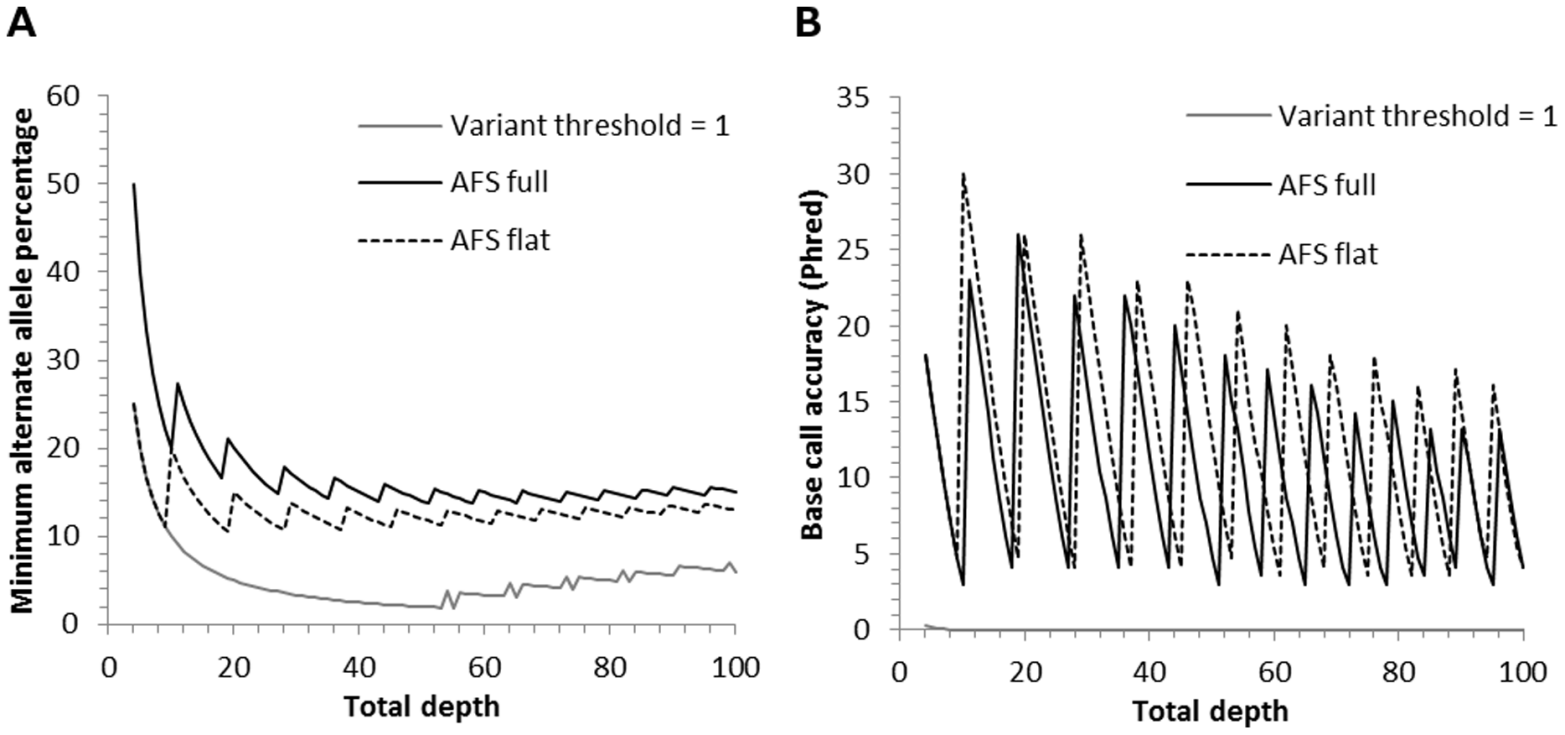
Evaluation of select methods of exome analysis using simulated data. (A) Minimum mutant allele frequencies for identification of a simulated variant at total read depths ranging from 4 to 100. (B) Base call accuracies of a simulated variant identified at the minimum mutant allele frequency. As total depth increases, the minimum percentage of the alternative or mutant allele that is required to identify the variant decreases until a total depth is reached at which an additional read with the alternate allele is required to detect the variant. At these total read depths, the addition of an alternate read causes the mutant allele frequency and the base call accuracy to increase. Consequently, the plots of minimum alternate allele percentage (A) and corresponding base call accuracies (B) are composed of a series of spikes at which points an additional alternate read was required to identify the variant.

The sensitivity of our standard and alternative exome analysis pipelines to detect rare variants was further investigated by using simulated data to determine the minimum mutant allele frequency that was required for variant identification at total read depths ranging from 4 to 100. The flat AFS model identified the variant at mutant allele frequencies that were significantly lower than the default analysis (figure 2a, Wilcoxon signed rank test p = 1.8×10^−17^, AFS flat average 13% range 10.5–25% versus default average 16.9% range 13.7–50%). In addition, the base call accuracies of the AFS flat model were higher than the default analysis (figure 2b, AFS flat average phred = 12.5 range 3.54–30 versus default average phred = 10.6 range 3.01–26). The only method which identified the variant at frequencies that were lower than the flat AFS model was the variant threshold equal to 1. However, the resulting base call accuracies for this variant threshold are much lower than the other methods and indicate that this method may struggle to differentiate between sequencing errors and real variants (average phred = 0.006, range 0.27–4.8×10^−16^). The sensitivity and base calling accuracy of the remaining methods (AFS cond2, mutation rates equal to 0.01 and 0.1, and variant threshold equal to 0.75) were intermediate between the default and AFS flat options (supplementary figure S3).

In summary, the flat AFS model was determined to have the greatest power to detect variants with low mutant allele frequencies in whole exome data from 3 MEG cases and in simulated sequence data with a range of coverage and mutant frequencies. We therefore recommend that the flat AFS method is used in support of a standard pipeline when searching for causal mutation(s) that could have low levels of mutant reads as a result of somatic mutation. Since sequencing and alignment errors may also give rise to low frequency variants we also recommend that the flat AFS method is used in conjunction with stringent QC procedures.

## Discussion

Riviere et al [2] identified a striking correlation between MEG phenotypes and genotype to the extent that 23 out of 24 unrelated individuals with mutations in *PIK3CA* were diagnosed with MCAP and all 14 cases with MPPH, of which two are related, had a recurrent c1117G>A mutation in *PIK3R2* or a mutation in *AKT3*. The only exceptions were one individual with an *AKT3* mutation and an overlapping phenotype and one case with a *PIK3CA* mutation diagnosed with MPPH. On the basis of this correlation, patient 3 from our study with a *PIK3R2* mutation is expected to have MPPH. However, according to the phenotype of this patient and in particular the marked bifrontal band heterotopia (Table 1) this patient could be diagnosed with MCAP. Furthermore, syndactyly/polydactyly, which is a feature of MCAP, is not evident in patient 1 who has a *PIK3CA* mutation. Our findings suggest that there may be a greater degree of overlap between these phenotypic classifications than the available studies suggest.

The receptor tyrosine kinase (RTK)-PI3K-AKT pathway is known to promote growth and contain additional disease related somatic mutations [12]. Other sporadic disorders caused by somatic mosaic events rather than germline mutations include activating mutations of *AKT1* associated with Proteus syndrome [4] and gain of function mutations in *PIK3CA* causing macrodactyly [13]. Recently a syndrome comprising congenital lipomatous overgrowth, vascular malformations, epidermal nevi, and skeletal/spinal abnormalities (CLOVES syndrome) has been found to involve postzygotic mutations and identified somatic mosaic mutations in *PIK3CA* in affected individuals [14]. Exome sequencing revealed missense *PIK3CA* mutations in six patients with mutant allele frequencies that ranged 3% to 30%. To detect low frequency variants and discount false positive sequencing errors, the authors ranked variants according to the number of reads with the mutant allele hence a variant present in 3 of 50 reads (6%) was ranked higher than a variant present in one of 5 reads (20%). Similarly Riviere et al [2], after failing to identify causal mutations in some cases through their standard pipeline undertook a second analysis to examine all variants that did not meet their initial hard filtering criteria. They examined 6 case exomes and 174 controls and found many in-silico variants in *PIK3CA* which consisted of 1 or 2 reads with mutant alleles but only 12 variants that were supported by 3 or more mutant reads of which a significantly higher proportion were in the MCAP phenotype cohort. Using a threshold of four variant reads they identified candidate mosaic variants in five of the six cases and none in controls (confirmed as present by Sanger sequencing). This suggests that variants supported by one to three reads are sequencing artefacts although this is dependent on the depth of coverage. Furthermore, identification of low frequency variants using a threshold of four variant reads may be less practical when less is known about the causative pathways and many genes require screening.

We have found that the most effective strategy for identifying low level mutations, as applied to real and simulated sequence data, is to use an initial allele frequency spectrum (AFS) with a flat distribution for variant calling. In the observed whole exome data, the flat AFS provided the strongest support for a low level mosaic mutation which causes MCAP and was not detected by a standard method of analysis. Although a multi-sample method, which uses simultaneous assessment of multiple samples, also identified this low level mutation our results demonstrate that this is dependent on the same variant being present at a high mutation frequency in at least one other sample. In addition our results indicate that, after stringent filtering, a larger number of apparently recurrent variants remained when using the multi-sample approach which could impede identification of causal variation in other samples. We also detected the low level mutation using substantial alteration of the variant threshold or the scaled mutation rate. However, both of these methods gave poor base calling accuracy and objective selection of appropriate thresholds is more challenging than adopting the flat AFS model when searching for low frequency mutations. These findings were replicated and expanded by the analysis of simulated data which showed that the flat AFS model identified variants at significantly lower mutation frequencies and higher base call accuracies than the default method (AFS full) at a range of sequencing depths.

### Ethics Statement

The patients were seen as part of the NHS clinical diagnositc service and we have written consent from the parents of the patients for the collection of DNA, analysis, and publication of results including a description of their children’s symptoms. In order to investigate the cause of their disorder, an exome sequencing approach was used after negative results were obtained from other diagnostic tests. This strategy is in accordance with the patients referral to clinical diagnostic and the wishes of the patients parents to determine the cause of their children's syndrome. When the samples were sent for exoming and analysis, codes only were used rather than patient identifiers. Once the findings were established the work was confirmed in a NHS accredited lab and the genetic diagnoses were given to the patients. However, this ethical consent does not permit public release of the patients whole exome sequence data.

## Supporting Information

Figure S1 **Variants identified in sample 1 by the full and flat AFS models including the causal post zygotic variant in** ***PIK3CA.***
(TIF)

Figure S2 **Variants identified in sample 3 by the full and flat AFS models including the causal variant in** ***PIK3R2.***
(TIF)

Figure S3 **Evaluation of all exome analyses using simulated data.** (A) Minimum mutant allele frequencies for identification of a simulated variant at total read depths ranging from 4 to 100. (B) Base call accuracies of a simulated variant identified at the minimum mutant allele frequency.
(TIF)

Table S1 **Coverage statistic.**
(DOCX)

Table S2 **Sequence and data quality characteristics of tier 1 variants identified by the default and alternative exome analyses.**
(DOCX)

Table S3 **Variant details for genes with novel tier 2 variants in two or more patients.**
(DOCX)

Table S4 **Number and sequence properties of unique tier 2 identified by the AFS flat model.**
(DOCX)
