# Supplementary figures and images for: Megalencephaly Syndromes: Exome Pipeline Strategies for Detecting Low-Level Mosaic Mutations
Source: PLoS One. 2014 Jan 31;9(1):e86940. doi: 10.1371/journal.pone.0086940 (PMC3908952; doi:10.1371/journal.pone.0086940)

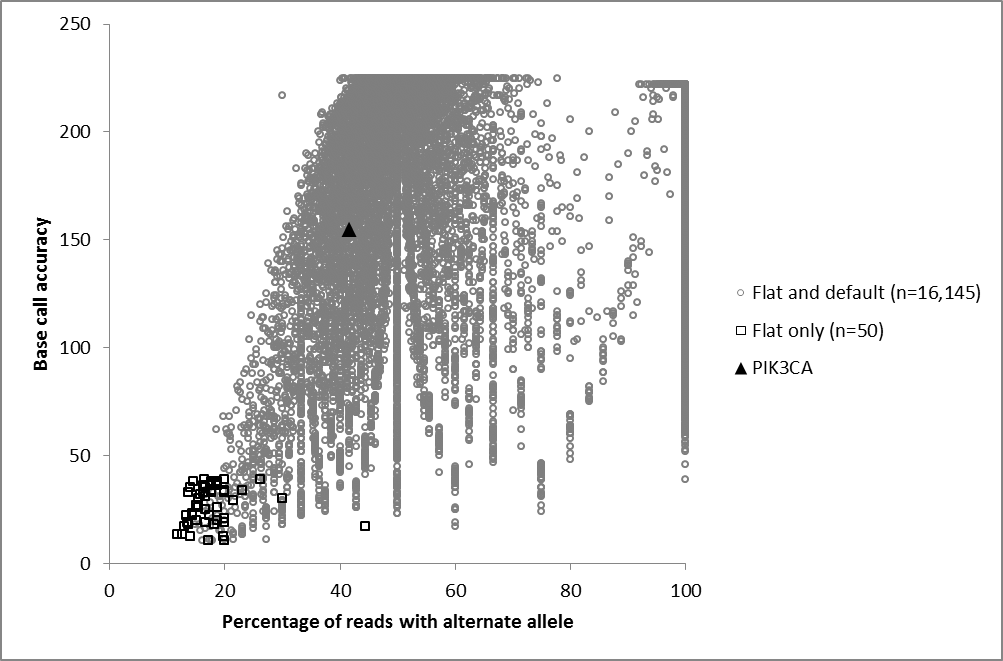

Supplement: Figure S1 — Variants identified in sample 1 by the full and flat AFS models including the causal post zygotic variant in PIK3CA. (TIF) [file pone.0086940.s001.tif]

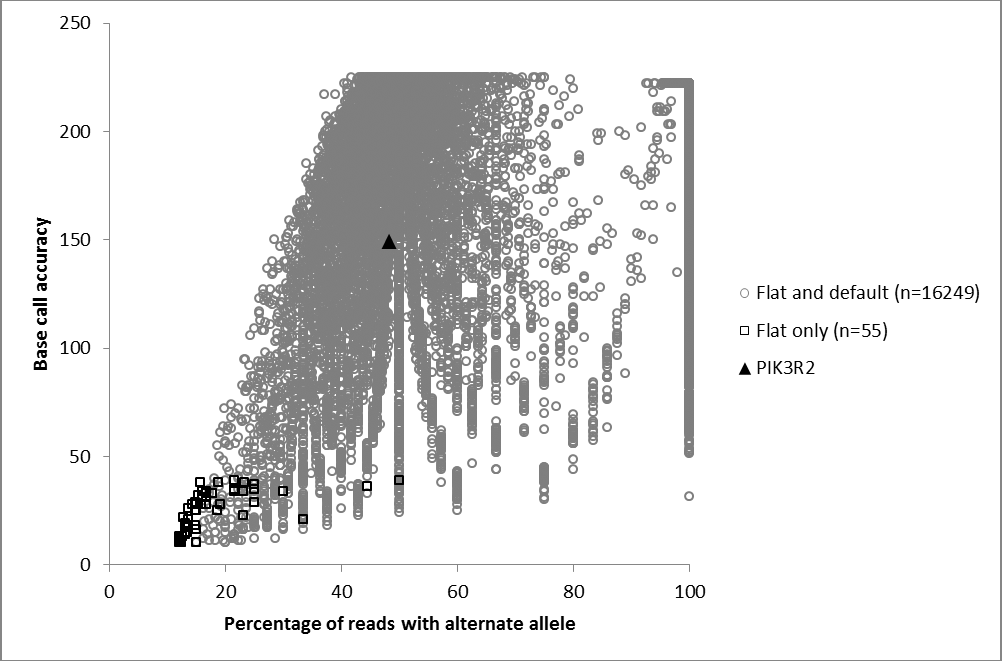

Supplement: Figure S2 — Variants identified in sample 3 by the full and flat AFS models including the causal variant in PIK3R2. (TIF) [file pone.0086940.s002.tif]

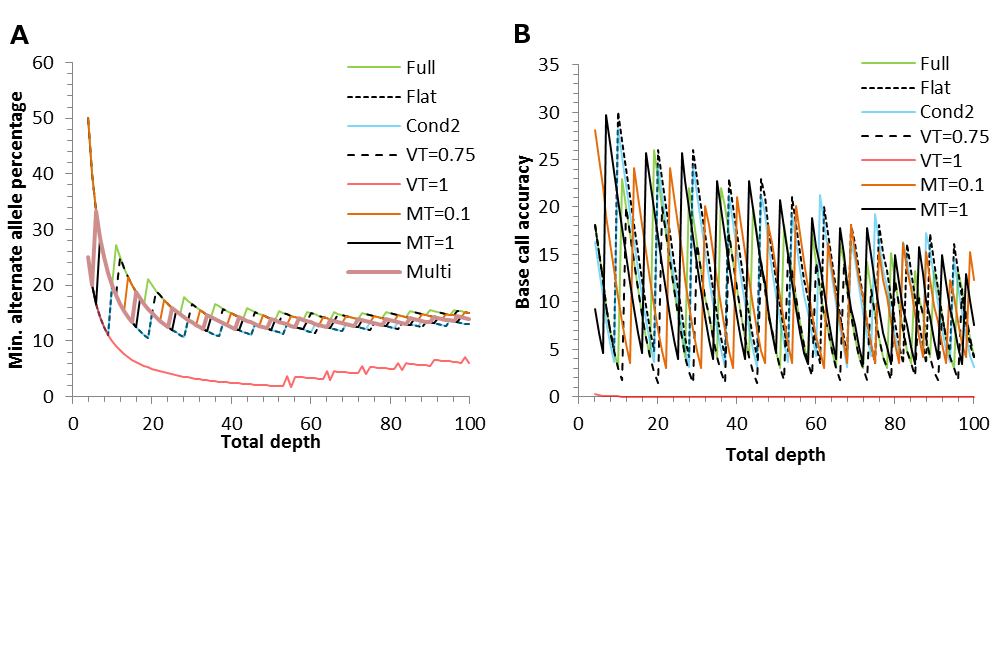

Supplement: Figure S3 — Evaluation of all exome analyses using simulated data. (A) Minimum mutant allele frequencies for identification of a simulated variant at total read depths ranging from 4 to 100. (B) Base call accuracies of a simulated variant identified at the minimum mutant allele frequency. (TIF) [file pone.0086940.s003.tif]
