# Supplementary material for: Megalencephaly Syndromes: Exome Pipeline Strategies for Detecting Low-Level Mosaic Mutations
Source: PLoS One. 2014 Jan 31;9(1):e86940. doi: 10.1371/journal.pone.0086940 (PMC3908952; doi:10.1371/journal.pone.0086940)
Supplement: Table S1 — Coverage statistic. (DOCX) [file pone.0086940.s004.docx]

**Table S1. Coverage statistic**

| Coverage statistic | Sample | | |
| --- | --- | --- | --- |
|  | 1 | 2 | 3 |
| Number of read sequences | 55,881,086 | 49,289,932 | 52,326,940 |
| Number of aligned read sequences | 54,998,471 | 48,403,517 | 51,374,345 |
| Number of reads with unique alignment | 50,153,761 | 44,577,371 | 47,442,927 |
| % of reads mapped to target | 66.03 | 63.07 | 63.18 |
| % of reads mapped to target ±150bp | 72.84 | 70.39 | 70.01 |
| % of target bases covered by ≥1 reads | 97.96 | 97.79 | 97.83 |
| % of target bases covered by ≥5 reads | 92.20 | 91.20 | 91.64 |
| % of target bases covered by ≥10 reads | 86.94 | 85.04 | 85.86 |
| % of target bases covered by ≥20 reads | 76.52 | 73.37 | 74.91 |
| Mean read depth | 51.85 | 46.26 | 48.40 |

Number of reads recorded as: (reference +ve strand, reference -ve strand, alternative +ve strand, alternative -ve strand)
