# Supplementary material for: Megalencephaly Syndromes: Exome Pipeline Strategies for Detecting Low-Level Mosaic Mutations
Source: PLoS One. 2014 Jan 31;9(1):e86940. doi: 10.1371/journal.pone.0086940 (PMC3908952; doi:10.1371/journal.pone.0086940)
Supplement: Table S2 — Sequence and data quality characteristics of tier 1 variants identified by the default and alternative exome analyses. (DOCX) [file pone.0086940.s005.docx]

**Table S2. Sequence and data quality characteristics of tier 1 variants identified by the default and alternative exome analyses**

|  | Mean number of variants | Mean number of novel variants | Mean % of variants in highly mutable genes | Mean % of variants with biases (P≤0.01) | Mean % of variants with 2 mutant reads | Mean % of unistranded variants | Mean base call accuracy (Phred) | Mean depth | Mean number of mutant reads | Mean % of mutant reads | Mean % of mutant reads in heterozygotes |
| --- | --- | --- | --- | --- | --- | --- | --- | --- | --- | --- | --- |
| Default | 22275 | 191 | 13.4 | 11.5 | 1.0 | 5.6 | 196.8 | 45 | 29 | 9.7 | 47.0 |
| Unique to AFS flat versus default | 1283 | 507 | 17.8 | 28.8 | 63.1 | 48.6 | 24.1 | 21 | 4 | 7.1 | 22.4 |
| Unique to AFS cond2 versus default | 1185 | 448 | 18.2 | 27.7 | 62.4 | 48.1 | 23.5 | 21 | 4 | 7.1 | 22.8 |
| Unique to mutation rate of 0.01 versus default | 410 | 87 | 20.1 | 19.5 | 61.1 | 51.5 | 15.0 | 19 | 4 | 11.1 | 27.6 |
| Unique to mutation rate of 0.1 versus default | 859 | 277 | 19.1 | 24.2 | 61.8 | 49.3 | 19.9 | 21 | 4 | 7.1 | 24.4 |
| Present in multi-sample and default | 21897 | 158 | 13.0 | 20.8 | 0.2 | 2.6 | 768.9 | 46 | 24 | 1 | 37.1 |
| Unique to multi-sample versus default | 2036 | 47 | 14.2 | 16.4 | 10.4 | 31.9 | 168.6 | 10 | 3 | 0.9 | 40.1 |
| Present in default absent from multi-sample | 378 | 32 | 33.6 | 47.4 | 16.0 | 37.1 | 76.5 | 51.9 | 20.8 | 11.1 | 37.4 |
