# Supplementary material for: Megalencephaly Syndromes: Exome Pipeline Strategies for Detecting Low-Level Mosaic Mutations
Source: PLoS One. 2014 Jan 31;9(1):e86940. doi: 10.1371/journal.pone.0086940 (PMC3908952; doi:10.1371/journal.pone.0086940)
Supplement: Table S3 — Variant details for genes with novel tier 2 variants in two or more patients. (DOCX) [file pone.0086940.s006.docx]

**Table S3. Variant details for genes with novel tier 2 variants in two or more patients**

| **Method** | **Sample** | **Reference/Alternative allele** | **Genotype** | **Gene** | **Variant type** | **Variant info** | **phylop** | **1-sift** | **polyphen2** | **Lrt** | **mutationtaster** | **gerp++** | **Grantham** | **Base call accuracy (Phred)** | **Number of reads** | **Phred-scaled Genotype likelihoods** | **Genotype quality (Phred)** |
| --- | --- | --- | --- | --- | --- | --- | --- | --- | --- | --- | --- | --- | --- | --- | --- | --- | --- |
| Multi-sample | 1 | GGACGAGCAGGATTGGA/- | HOM | AURKC | Splicing | N/A |  |  |  |  |  |  |  | 139 | 0,0,1,2 | 120,6,0 | 10 |
| Multi-sample | 2 | GGACGAGCAGGATTGGA/- | HOM | AURKC | Splicing | N/A |  |  |  |  |  |  |  | 139 | 0,0,1,2 | 60,3,0 | 7 |
| Multi-sample | 3 | GGACGAGCAGGATTGGA/- | HOM | AURKC | Splicing | N/A |  |  |  |  |  |  |  | 139 | 0,0,1,2 | 0,0,0 | 5 |
| Multi-sample | 1 | CC/- | HOM | NPEPL1 | frameshift deletion | c.42_43del:p.14_15del |  |  |  |  |  |  |  | 73.7 | 0,0,3,2 | 114,15,0 | 17 |
| Multi-sample | 2 | CC/- | HOM | NPEPL1 | frameshift deletion | c.42_43del:p.14_15del |  |  |  |  |  |  |  | 73.7 | 0,0,3,2 | 0,0,0 | 4 |
| Multi-sample | 3 | CC/- | HOM | NPEPL1 | frameshift deletion | c.42_43del:p.14_15del |  |  |  |  |  |  |  | 73.7 | 0,0,3,2 | 0,0,0 | 4 |
| Multi-sample | 1 | T/C | HET | TTLL5 | Synonymous | c.T738C:p.A246A |  |  |  |  |  |  |  | 999 | 29,58,14,11 | 246,0,205 | 99 |
| Multi-sample | 2 | A/G | HET | TTLL5 | nonsynonymous | c.A53G:p.D18G | 0.997 | 0.99 | 0.924 | 0.95 | 0.39161 | 4.76 | 94 | 120 | 17,26,3,7 | 155,0,183 | 99 |
| All | 2 | C/T | HET | BAHCC1 | Synonymous | c.C399T:p.A133A |  |  |  |  |  |  |  | 128 | 1,4,1,7 | 128,0,83 | 86 |
| All | 3 | C/T | HET | BAHCC1 | Synonymous | c.C5817T:p.A1939A |  |  |  |  |  |  |  | 157 | 1,5,4,5 | 157,0,93 | 96 |
| All | 2 | C/T | HET | DIP2C | Splicing | c.3584+5G>A |  |  |  |  |  |  |  | 175 | 12,5,11,3 | 175,0,200 | 99 |
| All | 3 | G/A | HET | DIP2C | nonsynonymous | c.C950T:p.P317L | 0.999 | 1 | 0.999 | 1 | 0.999957 | 4.63 | 98 | 147 | 5,7,5,5 | 147,0,170 | 99 |
| All | 1 | C/T | HET | KIAA1324 | Synonymous | c.C2448T:p.I816I |  |  |  |  |  |  |  | 191 | 3,4,7,5 | 191,0,115 | 99 |
| All | 2 | G/A | HET | KIAA1324 | nonsynonymous | c.G1705A:p.V569I | 0.992 | 0.34 | 0.339 | 0.98 | 0 | 3.83 | 29 | 255 | 21,11,17,18 | 255,0,230 | 99 |
| All | 1 | G/A | HET | PIK3CA | nonsynonymous | c.G2176A:p.E726K | 0.999 | 0.78 | 0.16 | 0.99 | 0.982342 | 5.02 | 56 | 185 | 8,13,4,11 | 185,0,236 | 99 |
| Flat, cond2, multi-sample | 2 | G/A | HET | PIK3CA | nonsynonymous | c.G2176A:p.E726K | 0.999 | 0.78 | 0.16 | 0.99 | 0.982342 | 5.02 | 56 | 12.3 | 13,19,1,3 | 12,0,233 | 15 |
| All | 1 | G/C | HET | PLEKHG5 | Splicing | c.1681-9C>G |  |  |  |  |  |  |  | 108 | 2,4,3,3 | 108,0,113 | 99 |
| All | 3 | C/T | HET | PLEKHG5 | nonsynonymous | c.G823A:p.G275S | 0.803 | 0.32 | 0 | 0.99 | 0.002697 | -0.431 | 56 | 89 | 1,1,2,2 | 89,0,42 | 45 |
| All | 1 | T/A | HET | TP53BP1 | nonsynonymous | c.A3520T:p.I1174L | 0.224 | 0.65 | 0 | 0.87 | 5.00E-04 | -0.612 | 5 | 220 | 18,19,12,11 | 220,0,255 | 99 |
| All | 3 | G/A | HET | TP53BP1 | nonsynonymous | c.C4037T:p.P1346L | 0.999 | 0.98 | 0.616 | 0.99 | 0.00211 | 4.11 | 98 | 233 | 9,5,13,8 | 233,0,175 | 99 |

Number of reads recorded as: (reference +ve strand, reference -ve strand, alternative +ve strand, alternative -ve strand)
