# Supplementary material for: Megalencephaly Syndromes: Exome Pipeline Strategies for Detecting Low-Level Mosaic Mutations
Source: PLoS One. 2014 Jan 31;9(1):e86940. doi: 10.1371/journal.pone.0086940 (PMC3908952; doi:10.1371/journal.pone.0086940)
Supplement: Table S4 — Number and sequence properties of unique tier 2 identified by the AFS flat model. (DOCX) [file pone.0086940.s007.docx]

**Table S4. Number and sequence properties of unique tier 2 identified by the AFS flat model**

| Sample | Total number of unique variants | Number of variants with ≤20% mutant reads | Mean and range of mutant reads % | Base call accuracy |
| --- | --- | --- | --- | --- |
| 1 | 50 | 46 | 17.8 (11.8 – 44.4) | 27.2 (10.4 – 39) |
| 2 | 28 | 24 | 17.6 (11.1 – 50.0) | 29.7 (12.3 – 39) |
| 3 | 55 | 42 | 17.8 (9.3 – 50.0) | 25.5 (10.4 – 39) |
| Mean | 44 | 37 | 17.7 | 27.5 |
